# Extracellular Trap Formation in Response to *Trypanosoma cruzi* Infection in Granulocytes Isolated From Dogs and Common Opossums, Natural Reservoir Hosts

**DOI:** 10.3389/fmicb.2018.00966

**Published:** 2018-05-15

**Authors:** Nicole de Buhr, Marta C. Bonilla, Mauricio Jimenez-Soto, Maren von Köckritz-Blickwede, Gaby Dolz

**Affiliations:** ^1^Department of Physiological Chemistry and Infectious Diseases, University of Veterinary Medicine Hannover, Hannover, Germany; ^2^Research Center for Emerging Infections and Zoonoses (RIZ), University of Veterinary Medicine Hannover, Hannover, Germany; ^3^Escuela de Medicina Veterinaria, Universidad Nacional, Heredia, Costa Rica

**Keywords:** *Trypanosoma cruzi*, common opossum, dog, ETs, granulocytes, immune system

## Abstract

Granulocytes mediate the first line of defense against infectious diseases in humans as well as animals and they are well known as multitasking cells. They can mediate antimicrobial activity by different strategies depending on the pathogen they encounter. Besides phagocytosis, a key strategy against extracellular pathogens is the formation of extracellular traps (ETs). Those ETs mainly consist of DNA decorated with antimicrobial components and mediate entrapment of various pathogens. In the last years, various studies described ET formation as response to bacteria, viruses and parasites e.g., *Trypanosma (T.) cruzi*. Nevertheless, it is not fully understood, if ET formation helps the immune system to eliminate intracellular parasites. The goal of this study was to analyze ET formation in response to the intracellular parasite *Trypanosma (T.) cruzi* by granulocytes derived from animals that serve as natural reservoir. Thus, we investigated the ET formation in two *T. cruzi* reservoirs, namely dogs as domestic animal and common opossums (*Didelphis marsupialis*) as wild animal. Granulocytes were harvested from fresh blood by density gradient centrifugation and afterwards incubated with *T. cruzi*. We conducted the analysis by determination of free DNA and immunofluorescence microscopy. Using both methods, we show that *T. cruzi* efficiently induces ET formation in granulocytes derived from common opossum as well as dog blood. Most ETs from both animal species as response to *T. cruzi* are decorated with the protease neutrophil elastase. Since *T. cruzi* is well known to circulate over years in both analyzed animals as reservoirs, it may be assumed that *T. cruzi* efficiently evades ET-mediated killing in those animals. Therefore, ETs may not play a major role in efficient elimination of the pathogen from the blood of dogs or common opossums as *T. cruzi* survives in niches of their body. The characterization of granulocytes in various animals and humans may be helpful to understand the anti-pathogenic capacity and overall role of ETs against zoonotic pathogens like *T. cruzi*.

## Introduction

The first line of defense against invading pathogens in humans and animals is formed by granulocytes that are able to rapidly infiltrate the infected tissue. All granulocytes are well known as multitasking cells and can mediate antimicrobial activity by various strategies e.g., intracellular digestion (phagocytosis) or degranulation of proinflammatory cytokines. In 2004, extracellular trap (ET) formation as an occurrence of a distinct extracellular off-shoot of the cells was reported as a fundamental new defense mechanism against various pathogens in cells of the innate immune system. These structures are mainly formed by neutrophil granulocytes and therefore often named as neutrophil extracellular traps (NETs) (Brinkmann et al., 2004), but additional evidence is also showing ET release by other granulocytes e.g., eosinophils as reviewed by Goldmann and Medina (2012). Those ETs or NETs consist of DNA and histones decorated with granule proteins as cell-type-specific proteases (e.g., elastase) (Urban et al., 2009) and cationic antimicrobial peptides (Neumann et al., 2014) that mediate extracellular entrapment and partial killing of microbes. It seems that ET formation is an old evolutionary mechanism as it could be detected so far in different species, such as human, mouse, cat, cattle, pig and fish (Brinkmann et al., 2004; Ermert et al., 2009; Wardini et al., 2010; Brogden et al., 2012; de Buhr et al., 2014; de Buhr and von Köckritz-Blickwede, 2016; Jerjomiceva et al., 2016) and in plants (Hawes et al., 2011; Tran et al., 2016). Furthermore, in 2015 the first publication about ETs in dogs was published (Jeffery et al., 2015), but only limited information is available about ET release in wild animals (Reichel et al., 2015). Interestingly, granulocytes of humans and animal species react differently after treatment with the same stimulus. For example porcine and human neutrophils release NETs after treatment with the Gram positive bacteria *Streptococcus suis* or the chemical stimuli phorbol-myristate-acetate in different amount (de Buhr et al., 2014). Furthermore, differences in NET formation of bovine and ovine neutrophils in response to *Toxoplasma gondii* were identified. Thus, it was discussed if maybe ET formation is involved in the host susceptibility to infection (Yildiz et al., 2017). From the point of view that zoonotic agents infect humans and animals and can induce varying symptoms depending on the species that is infected, a comparable closer look to the immune responses could help to understand the species-specific pathogenesis of zoonotic diseases. ETs have been well described as response to bacteria, but only little until now on ETs and parasites. ET formation was described in response to *Toxoplasma gondii* (Abi Abdallah et al., 2012; Yildiz et al., 2017), *Plasmodium falciparum, Eimeria bovis, Leishmania amazonensis, Leishmania chagasi, Leishmania donovani*, and *Leishmania major* (reviewed by Abi Abdallah and Denkers, 2012). Recently a study reported a decreased NET release in presence of *Leishmania infantum* by canine neutrophils (Pereira et al., 2017), whereas *Leishmania* species induce NETs in human neutrophils (Guimarães-Costa et al., 2009; Gabriel et al., 2010). Interestingly, the zoonotic pathogen *Trypanosoma (T.) cruzi* has been demonstrated to induce NETs in human neutrophils and thus NETs have been discussed to limit infection (Sousa-Rocha et al., 2015). In mouse experiments, pre-treatment of parasites with NETs resulted in a significantly decreased number of parasites in the blood (Sousa-Rocha et al., 2015).

*T. cruzi* leads to Chagas disease or American trypanosomiasis and is described by the World Health Organization (WHO) as a worldwide existing major neglected disease. Up to 7 million people worldwide are estimated to be infected (World Health Organization (WHO), 2017). As *T. cruzi* infects numerous mammals, it is interesting how the immune system of carriers reacts during infection. Natural carriers are for example sheep (*Ovis aries*), cats (*Felis silvestris catus*), dogs (*Canis familiaris*), mice (*Mus musculus*), rats (*Rattus rattus*), and opossums (*Didelphis marsupialis*) (reviewed by Álvarez-Hernández et al., 2016). Transmission cycle studies and other studies described the domestic dog as a natural host in domestic or peridomestic areas. Furthermore the opossum is described as a major reservoir in sylvatic areas (reviewed by Fernandes et al., 1991; Travi et al., 1994; Gürtler and Cardinal, 2015). Interestingly, opossums give birth to offspring with an undeveloped immune system and it was discussed that the didelphine immune system can help to understand the evolution of the mammalian immune system (Belov et al., 2007). Already in 1988 a specific host-parasite relationship between opossums and *T. cruzi* was discussed (Jansen, 1988).

In this study we aimed to investigate if dog and common opossum granulocytes release ETs as response to the parasite *T. cruzi* as it was previously described for human granulocytes. It is known that besides dogs also opossums are natural reservoirs of *T. cruzi* in a high prevalence (Jansen et al., 1991; Araujo Carreira et al., 1996). Thus, it may be hypothesized that granulocytes of dogs and opossums as natural reservoir are hampered to be involved in the clearance of *T. cruzi*. Therefore, we were wondering if granulocytes derived from opossum and dogs eventually lack the ability to release ETs.

## Results and discussion

### Presence of extracellular DNA in canine and didelphine blood

In this study we aimed to investigate the formation of ETs by granulocytes from dogs (*Canis lupus familaris*) and the common opossum (*Didelphis (D.) marsupialis*). The dogs included in this study were kept in the houses close to their owners in Heredia, Costa Rica. The common opossums included in this study were trapped close to the canteen of the Escuela de Medicina Veterinaria, Universidad Nacional, Heredia, Costa Rica, as a wild animal living in neighborhood to humans.

As all animals included in this study were potentially *T. cruzi* infected, we tested the blood of all of them with nested PCR 18S rRNA analysis. All common opossums were positive tested (GenBank accession numbers MH020165, MH020166 MH020167, MH020168, and MH020169) and all dogs were negative tested (Supplementary Figure 1).

**Figure 1:**
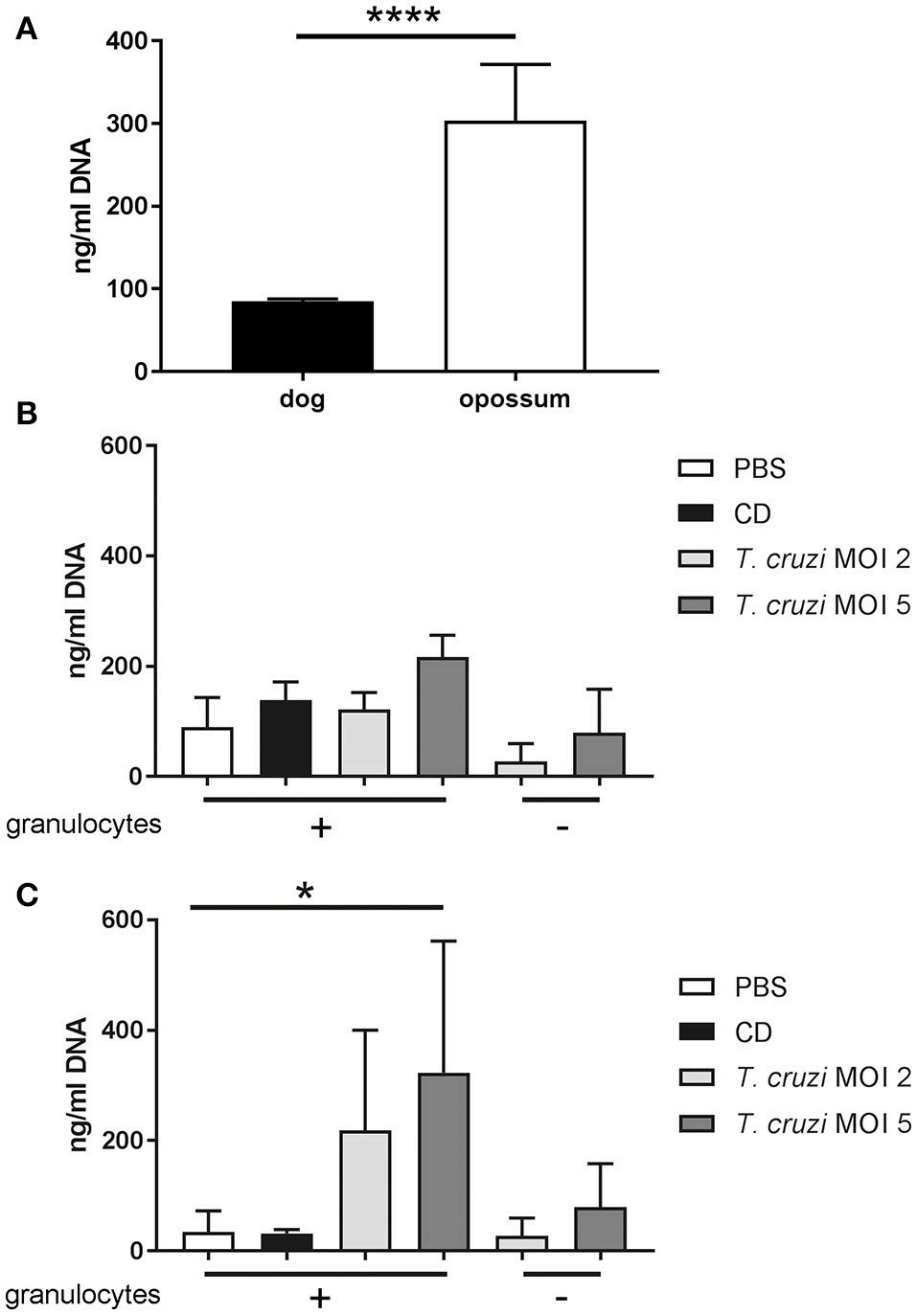
Free DNA is released from canine and didelphine granulocytes after *T. cruzi* infection. **(A)** Free DNA was measured in plasma of heparin blood after 2 h incubation at 37°C, 5% CO_2_ (1:2 diluted with PBS). Significant higher amount of free DNA was detectable in opossum plasma by unpaired, two-tailed Student's *t*-Test (*n* = 5, ^****^P < 0.0001). **(B,C)** Isolated granulocytes were incubated for 3 h with PBS as negative control and the cholesterol-depleting cyclodextrine (CD) as positive ET inducer or *T. cruzi* with MOI 2 or 5. Afterwards free DNA in supernatants as an indicator for ETs was measured with Pico Green test. **(A)** No significant difference was detected in the analyzed dogs by Friedman test (*n* = 3). **(B)** No significant difference was detected in the analyzed dogs by Friedman test (*n* = 3). **(C)** In contrast a significant difference between negative control and *T. cruzi* MOI 5 was detected in the analyzed opossums by Friedman test (*P* = 0.0174) followed by Dunn's multiple comparisons test (*P* = 0.0342) (*n* = 3, ^*^P < 0.05).

To get a general overview about differences in the reaction of the blood from both species, we incubated heparin blood with PBS (1:2 diluted) for 2 h. Afterwards we measured free extracellular DNA in the plasma as a first screening to compare both species. Plasma free extracellular DNA has been considered as marker for ET-formation in blood (Margraf et al., 2008; Altrichter et al., 2010). Interestingly in the plasma of common opossums, significant more free DNA was detectable by Pico Green analysis (Figure 1A). Three explanations are reasonable: Firstly, common opossums have in general more free DNA in the blood compared to dogs, as maybe the immune system is all the time in a steady state stadium of activation e.g., since they are all positive for *T. cruzi* PCR. Secondly, the PCR detected infection with *T. cruzi* in the common opossums leads to the higher amount of free DNA derived from *T. cruzi* itself. Thirdly, the Pico Green assay is maybe not the best screening method, because the plasma color from common opossums (Supplementary Figure 2) can influence the fluorometric analysis. Based on this information and our recent review discussion about different methods to study ET formation (de Buhr and von Köckritz-Blickwede, 2016), we considered to perform ET assays with purified granulocytes rather than total blood.

### Comparison of purified granulocytes from canine and didelphine blood

To compare the granulocyte reaction of both animals on *T. cruzi*, we tried to undergo a comparable granulocyte isolation method with high amount of neutrophils as main granulocyte population. As the percentage of granulocytes in dog blood is higher than the lymphocyte number, they typically show a granulocytic blood count similar as human blood (von Engelhardt, 2015). But only little is known about the granulocyte number in opossums. A publication about *Didelphis virginiana* from 1980 report higher percentage of lymphocytes than granulocytes, thus, indicating that the opossum shows a lymphocytic blood count (Cutts and Krause, 1980). Looking to data from 2018 it seems that no clear blood count type is recognizable as the number of lymphocytes and granulocytes or neutrophils are more or less similar (Opossum Society of the United States, 2018). It was also mentioned that the cell numbers can vary between individual opossums and that eosinophil numbers up to 15% of white blood population are frequently seen. Nevertheless, most of the physiological neutrophil parameters of both animals are comparable (Table 1). For isolation of granulocytes we used the same isolation process for both species to avoid technical influences on the *in vitro* assays. The Biocoll density gradient of common opossums and dogs looked similar. Only small differences like thickness of the peripheral blood mononuclear cell layer and the plasma color were observed (Supplementary Figure 2). Interestingly, the isolation protocol seems to work more efficiently with the didelphine blood: The theoretical number (given by literature) of granulocytes in didelphine blood was very close to the indeed isolated number in our study. Distinctly less of the theoretical number of granulocytes was isolated in case of the blood from dogs (Supplementary Table 1). DIFF Quick staining of isolated granulocytes (Supplementary Figures 2D,F) revealed percentage of neutrophils, eosinophils and other cells in the isolated granulocyte population. Interestingly, the granulocyte fraction from opossum revealed 66% neutrophils and 33% eosinophils, whereas dog purity was 99% neutrophils (Supplementary Table 2). As all animals in this study were clinically healthy, the health status cannot explain the differences in the isolation efficiency. However, since the common opossum samples were positive for *T. cruzi* as tested by PCR (Supplementary Figure 1) that may explain the high number of eosinophils present in opossum. As all common opossums had to be anesthetized for the blood sampling and an influence of anesthesia cannot be excluded. To exclude influences of the blood taking procedure and isolation method on ET-formation in all assays the negative (unstimulated) as well as positive controls (ET-positive control) were included in the assays. Since eosinophils have been shown to release ETs with a similar function as neutrophils (reviewed by Goldmann and Medina, 2012), we kept eosinophils in our analysis.

**Table 1:** Overview about physiological neutrophil parameters of dogs and common opossums.

	**Dog (*Canis lupus familaris*)**	**Common opossum (*Didelphis marsupialis*)**
granulocytes/μl	3300–10200^*^	1800–8442^*^
eosinophils/μl	0–360	120–1890
neutrophils/μl	3300-9000^*^	1800–6300^*^
lobes nuclei/cell	multi-lobed	3
Myeloperoxidase	Yes	Yes
Able to form NETs	Yes (Jeffery et al., 2015)	Yes (Figure 3)
Special	relative high number of leukocytes in newborns	Very low number of neutrophils and less lobes in newborns (Cutts and Krause, 1980)
Cytoplasm	White, contains small pink granules	White with granula

* *von Engelhardt (2015) and Opossum Society of the United States (2018)*.

### Release of free extracellular DNA from didelphine granulocytes after *T. cruzi* infection

In the next step, we analyzed now if purified granulocytes from common opossums and dogs release free extracellular double-stranded DNA as an indicator for ETs. We investigated if a difference is detectable after stimulation with *T. cruzi*. Therefore, we isolated granulocytes freshly and stimulated them for 2 h with *T. cruzi*. In canine granulocytes, only slightly and not significant more free extracellular DNA was detected in the supernatants comparing negative control and *T. cruzi* (MOI 5) infected samples, especially after consideration of the *T. cruzi* MOI 5 background control signal (Figure 1B). Interestingly, the didelphine granulocytes released significant more free DNA after *T. cruzi* stimulation (MOI 5) (Figure 1C). Furthermore, the amount of free DNA in the PBS control was with a mean of 34.28 ng/ml in the didelphine sample lower compared to the canine sample with a mean of 89.7 ng/ml. This is a contrasting result compared to Figure 1A. An explanation could be that in general the granulocyte isolation method pre-stimulates canine granulocytes more than didelphine. Furthermore, in this assay the possible influence of the plasma color is not given.

It is of interest that inside the genome of *Monodelphis domestica* (Mikkelsen et al., 2007), which is phylogenetically close related to *D. marsupialis*, a very high number of antimicrobial peptides (AMPs) (12 cathelicidins and 32 beta-defensins, 1 alpha-defensin) was found (Belov et al., 2007). It was shown that the human AMP LL-37 diminish the DNA signal in the Pico Green assay (Neumann et al., 2014). Therefore, it can be hypothesized that maybe AMPs released in this assay by didelphine granulocytes also decrease the signal. Further studies are needed to identify if some of these AMPs are located in didelphine granulocytes. In dogs the presence of AMPs in neutrophils (Sang et al., 2007) have already been described and therefore here a comparable scenario may be hypothesized.

Nevertheless, the data indicate that didelphine granulocytes are stimulated by *T. cruzi* to release ETs which may be detectable in the supernatant as free extracellular DNA. However, the detected amount of free DNA reflects only free components. The DNA that is ET associated or intracellular is not detected with this method, as a centrifugation was included prior to DNA quantification. To analyse ET associated DNA without the problems in presence of AMP when performing the Pico Green assay, we confirmed the results by immunofluorescence microscopy as previously recommended in a review on visualization of ETs (de Buhr and von Köckritz-Blickwede, 2016).

### *T. cruzi* induces ETs in canine and didelphine granulocytes

**Figure 2:**
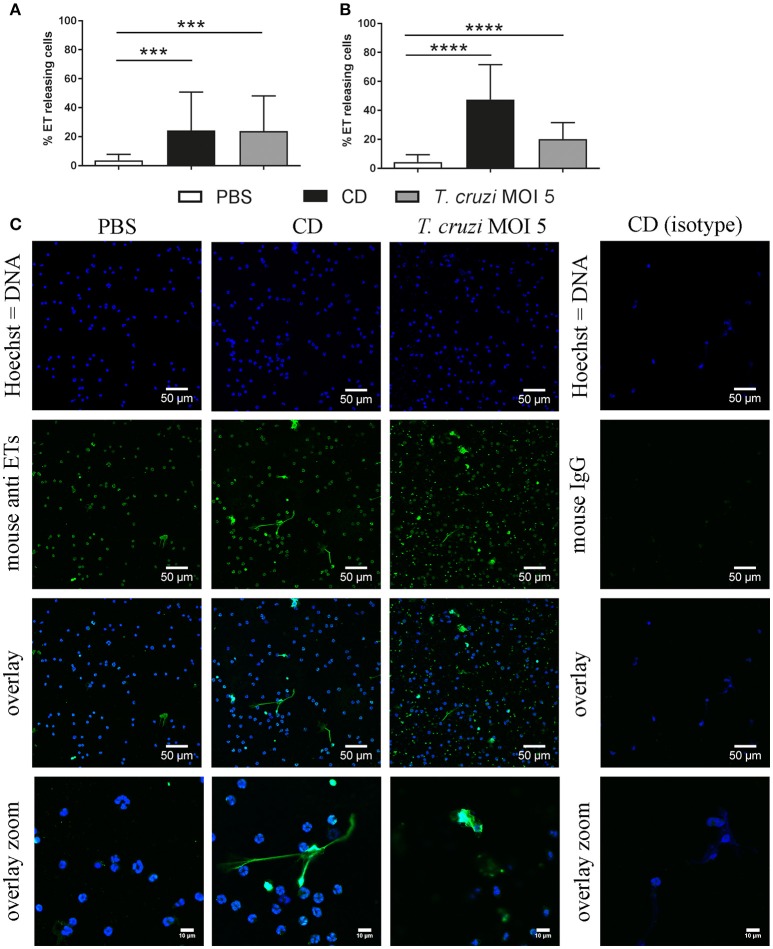
*T. cruzi* induces ETs in canine granulocytes *in vitro*. Formation of ETs after infection of purified canine granulocytes with *T. cruzi* at MOI of 5 after 60 min **(A)** and 180 min **(B)** was analyzed. PBS was used as negative control and the cholesterol-depleting cyclodextrine (CD) as positive ET inducer. After infection ETs were visualized by immunofluorescence microscopy (ETs [DNA/histone-1-complexes] = green, Hoechst [DNA] = blue). Per sample at least 6 pictures were taken at predefined positions, and the number of granulocytes and the ET-releasing cells were determined. **(C)** Representative pictures after 60 min of stimulation are shown. The isotype control was negative for ETs. Graphs in **(A,B)** show the mean ± SD of 3 independent experiments with 18 (180 min) or 19 (60 min) analyzed pictures. Statistical differences were tested by unpaired one-tailed Students *t*-Test (^***^P < 0.001; ^****^P < 0.0001).

**Figure 3:**
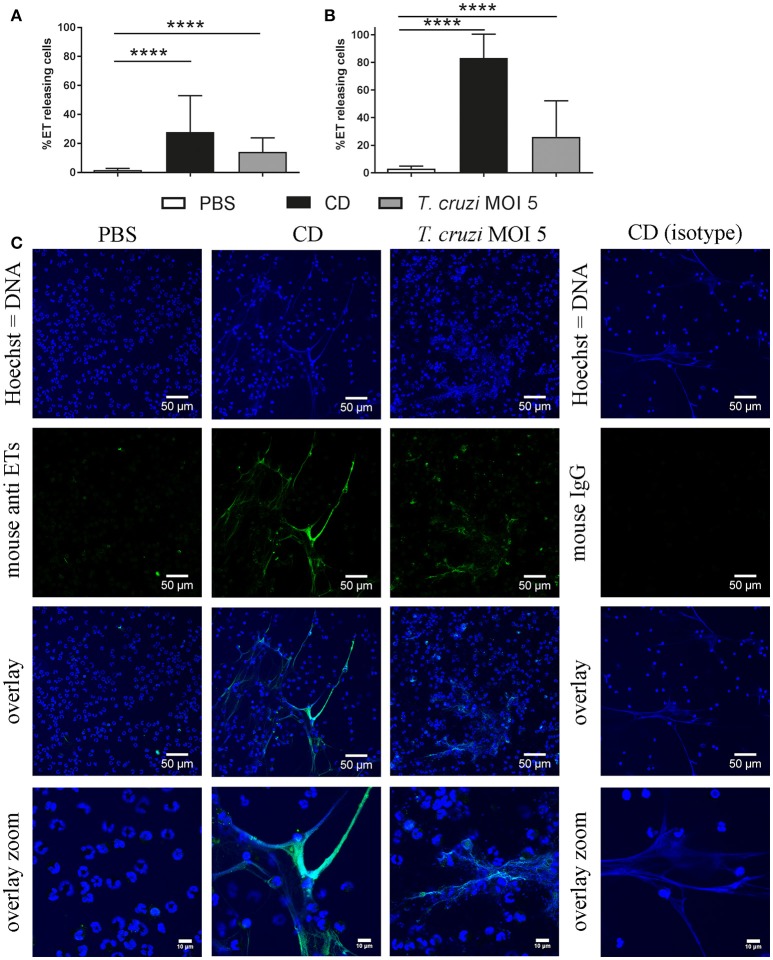
*T. cruzi* induces ETs in didelphine granulocytes *in vitro*. Formation of ETs after infection of purified didelphine granulocytes with *T. cruzi* at MOI of 5 after 60 min **(A)** and 180 min **(B)** was analyzed. PBS was used as negative control and the cholesterol-depleting cyclodextrine (CD) as positive ET inducer. After infection ETs were visualized by immunofluorescence microscopy (ETs [DNA/histone-1-complexes] = green, Hoechst [DNA] = blue). Per sample at least 6 pictures were taken at predefined positions, and the number of granulocytes and the ET-releasing granulocytes were determined. **(C)** Representative pictures after 60 min of stimulation are shown. The isotype control was negative for ETs. Graphs in A and B show the mean ± SD of 4 independent experiments with 24 analyzed pictures. Statistical differences were tested by unpaired one-tailed Students *t*-Test (^****^p < 0.0001).

Thus, to confirm the results that *T. cruzi* induces significantly more ETs in didelphine granulocytes compared to canine granulocytes, we performed an ET induction assay followed by immunofluorescence microscopy analysis to quantify ET formation as an occurrence of a distinct extracellular off-shoot of the cells which is positive for histone-DNA complexes. Purified canine and didelphine granulocytes were analyzed after same stimulation as described above over time. Since *T. cruzi* MOI 5 was shown to be the most efficient stimulus in the Pico Green analysis, we used this MOI for the next assay. In both species only a very low percentage of ET releasing cells were detectable in the negative control. Therefore, we concluded that the isolation method in both species is not significantly pre-stimulating the granulocytes (Figures 2, 3). In canine granulocytes the percentage of ET releasing cells increases over the time (60 min mean = 24.38%, 180 min mean = 47.47%) in the positive control incubated with the cholesterol depleting cyclodextrine (CD). In contrast to the result from Figure 2, here a significant difference between negative control and *T. cruzi* MOI 5 incubated sample was detectable indicating ET-formation as response to *T. cruzi* in canine granulocytes. But there is no further increase in ET releasing cells over time as found for the CD incubated sample (*T. cruzi* MOI 5: 60 min mean = 23.84%, 180 min mean = 20.17%; Figure 2). The ET release analysis with immunofluorescence microscope after *T. cruzi* incubation in didelphine granulocytes confirmed the Pico Green results shown in Figure 2 The didelphine granulocytes incubated with *T. cruzi* MOI 5 were releasing ETs similar to the canine granulocytes, but interestingly over the time an increase of ET-formation was detectable (*T. cruzi* MOI 5: 60 min mean = 14.13%, 180 min mean = 26.14%; Figure 3). But in contrast to the Pico Green results, here the positive control released high percentages of ETs that was increasing over time (CD: 60 min mean = 27.83%, 180 min mean = 83.17%; Figure 3). As shown in the images, the CD incubation resulted in very compacted ET structures in close contact to the granulocyte nuclei, which eventually may not be detected in the Pico Green assay. This could explain the difference results that less free components of DNA are detectable in the supernatant as shown in Figure 1.

**Figure 4:**
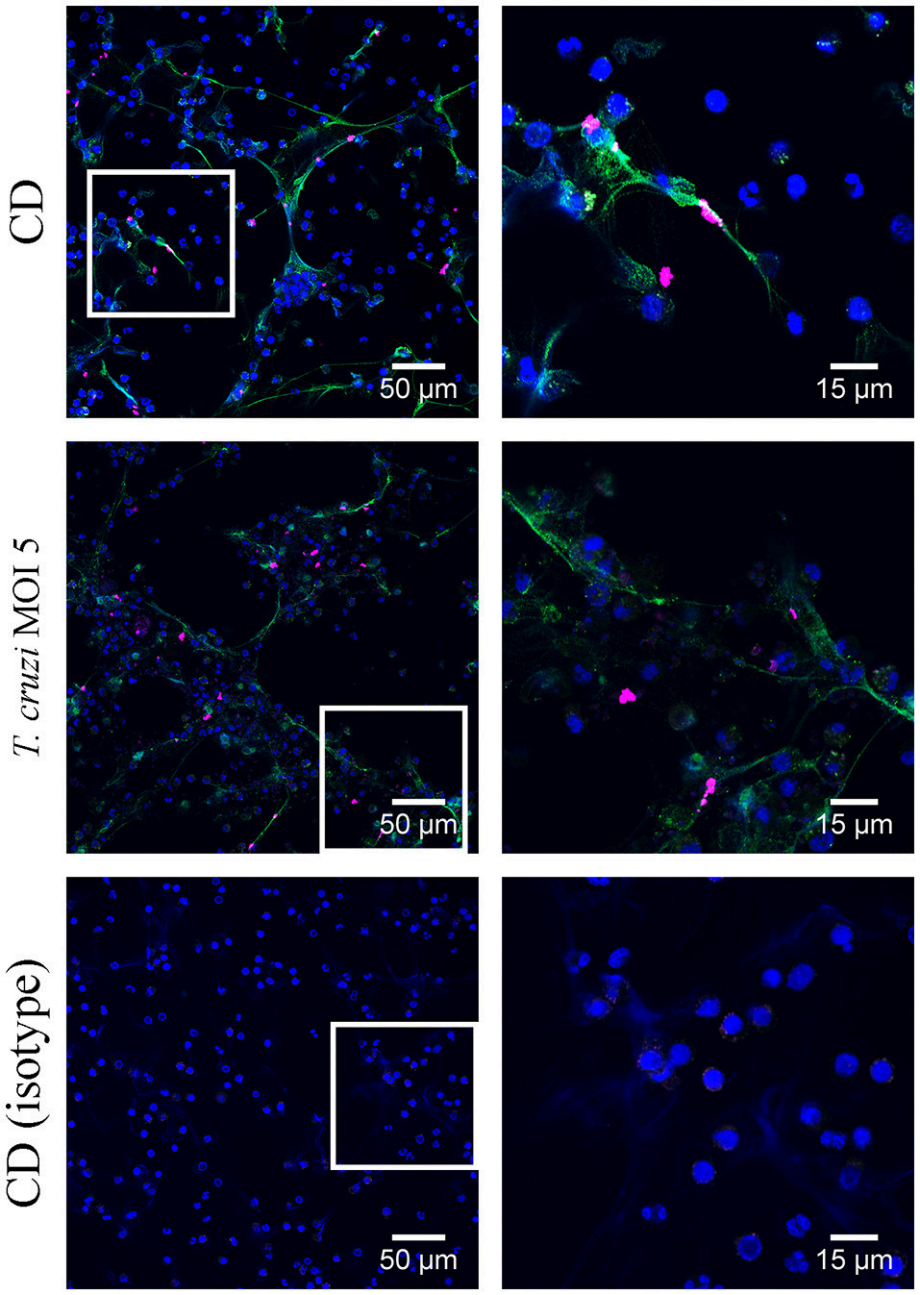
Neutrophil elastase detection in canine extracellular traps (ETs) Immunofluorescence microscopy allowed detection of neutrophil elastase (NE) in ETs after 180 min treatment with cholesterol-depleting cyclodextrine (CD) or *T. cruzi* (MOI = 5) as indicated. The isotype control was negative for NE and ETs. (blue = DNA [Hoechst], green = DNA/histone-1- complexes [ETs], pink = neutrophil elastase [NE]). Representative pictures are shown.

**Figure 5:**
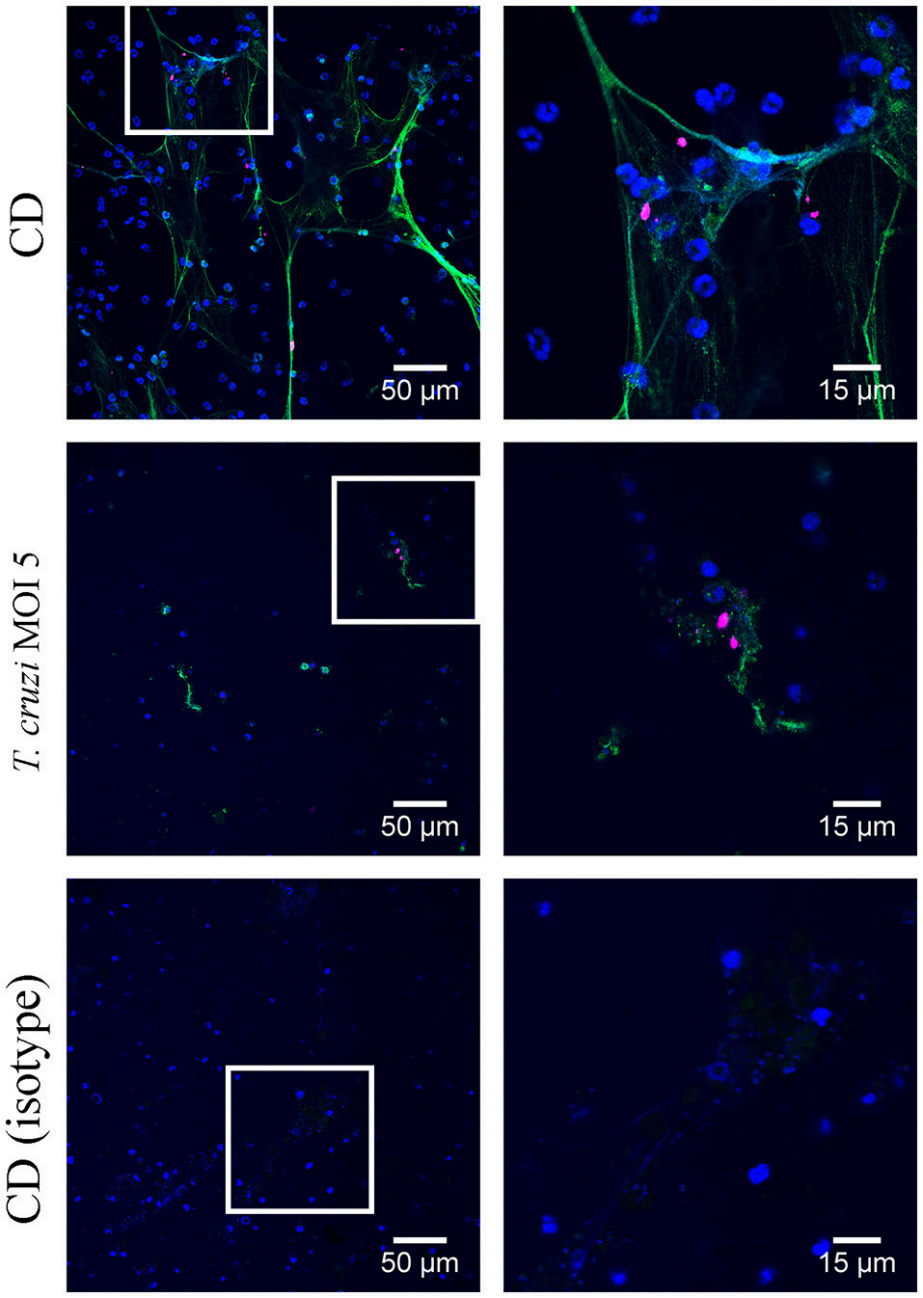
Neutrophil elastase detection in didelphine extracellular traps (ETs) Immunofluorescence microscopy allowed detection of neutrophil elastase (NE) in ETs after 180 min treatment with cholesterol-depleting cyclodextrine (CD) or *T. cruzi* (MOI = 5) as indicated. The isotype control was negative for NE and ETs. (blue = DNA [Hoechst], green = DNA/histone-1- complexes [ETs], pink = neutrophil elastase [NE]). Representative pictures are shown.

Bringing all results together, we conclude that canine and didelphine granulocytes release ETs after *T. cruzi* infection. However, the common opossum seems to react slightly stronger in contact to the pathogen as the amount of free DNA components is higher (Figure 1C) and in addition, the final percentage of ET releasing cells is slightly higher after 180 mins (Figure 2B, 3B). But if this slight difference explains different host susceptibility as described in case of ovine and bovine NETs and the *Toxoplasma gondii* infection (Yildiz et al., 2017) remains unclear and further studies are needed to answer this question. Based on the differences seen in eosinophil number (Supplementary Table 2), it might be speculated that the eosinophils contribute to a strong ET formation. Nevertheless, in both tested species most, but not all, ETs were positive for neutrophil elastase (NE) (Figures 4, 5), which is a prominent neutrophil granule component often associated with NETs and contributing to NET-mediating antimicrobial activity. It has to be mentioned, that the elastase-staining protocol was adjusted for staining of elastase within NET fibers, but not for complete intracellular staining of elastase in the granules.

**Figure 6:**
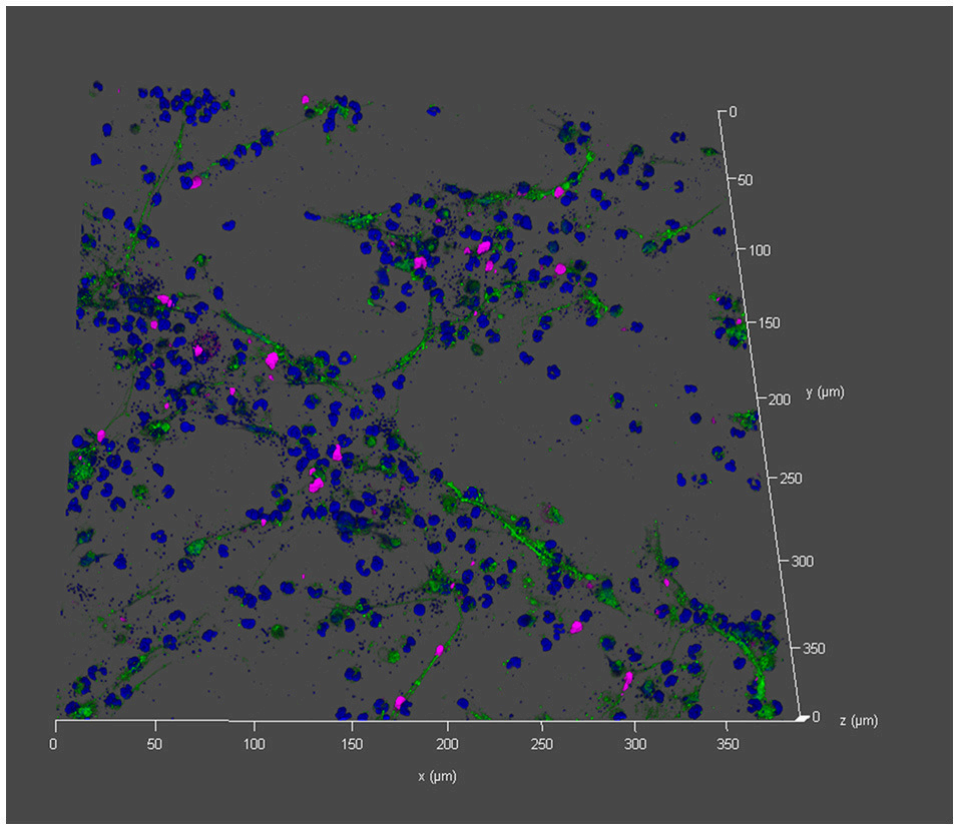
Entrapment of *T. cruzi* in didelphine ETs Immunofluorescence microscopy allowed detection of *T. cruzi* (blue small dots) localized in didelphine ETs after 180 min incubation (blue = DNA [Hoechst], green = DNA/histone-1- complexes [ETs], pink = neutrophil elastase [NE]). Less blue dots (*T. cruzi*) are detected outside the ET area. The isotype control was negative for NE and ETs. Out of a z-stack a 3D-constructed representative picture is shown.

Furthermore, an accumulation of *T. cruzi* inside the ETs area was visualized. 3D presentation showed only single *T. cruzi* outside the ET area (empty gray area) and *T. cruzi* as blue dots inside the ET area after 180 min incubation (Figure 6; Supplementary Video 1). Based on these results, we assume that granulocytes of opossum and dogs are not hampered in their ET-releasing activity.

In general it is known that dogs have a high prevalence to be naturally infected with *T. cruzi* by for example eating triatomines (reviewed by Teixeira et al., 2011). Serum positive naturally infected dogs undergo an acute phase, sometimes leading to death in young dogs. Furthermore, dogs can go over to a chronic phase. This phase is marked by cardiomyopathy symptoms like cardiomegaly or electrocardiogram (ECG) alterations and can lead to sudden death (Montenegro et al., 2002; Cruz-Chan et al., 2009). In addition naturally infected dogs develop hydrothorax, hydropericardium, edema or cyanosis which are all clinical and pathological manifestations described for other mammals and humans with Chagas disease (reviewed by Teixeira et al., 2011). Therefore, the question arises, how protective ETs are in dogs during acute *T. cruzi* infection and if they are maybe negatively involved in the development of chronic cardiomyopathy symptoms. As ETs are induced by *T. cruzi* they can also be present in the infected tissue. Interestingly DNA and ETs can have damaging effects on heart functions and increases myocardial ischemia in mice (Savchenko et al., 2014). Based on this hypothesis, we suggest investigating if ETs are found in the chronic phase of Chagas disease, and, thus to study if ETs contribute to detrimental effects.

In contrast, the *T. cruzi* infected opossums do not develop clinical signs of disease, but a detection of the parasite in the anal glands is possible (Deane et al., 1984; Legey et al., 2003). Furthermore, the parasite undergoes the vertebrate and invertebrate multiplication cycle in the opossum, thus *T. cruzi* is reacting in a different way in opossum compared to other mammals.

A possible explanation for species-specific susceptibility may be explained by species specific presence of host endogenous antimicrobial peptides. It was demonstrated that ancient AMPs from Australian marsupial kill antibiotic-resistant pathogens efficiently compared to human cathelicidin LL-37 (Wang et al., 2011). Several studies demonstrated the antimicrobial effect of AMPs from marsupial mammals and how these peptides protect the marsupial pouch (Cheng and Belov, 2017; Peel et al., 2017). An effective AMP could also help the opossum to fight against *T. cruzi* when trapped in ETs as known for LL-37 in humans or PR-39 in pigs (Neumann et al., 2014; de Buhr et al., 2017).

## Conclusion

Since *T. cruzi* is well known to circulate over years in both analyzed animals as reservoirs, it may be assumed that *T. cruzi* efficiently evades ET-mediated killing in dogs and opossum. Based on our data, that granulocytes from dogs and opossum release ETs as response to *T. cruzi*, we assume that ETs do not play a major role in elimination of the pathogen from the blood of dogs or common opossums as *T. cruzi* survives in niches of their body. ETs may indeed avoid spreading of the pathogen in different animal species but is not the only key factor to completely eliminate the pathogen from the blood.

Future work should focus on a better understanding if and how granulocytes act in concert with other components of the innate immune defense to contribute to a host-specific colonization and/or pathogenicity of zoonotic pathogens in various animal species.

However, the presented study with a future perspective may open new insights in the evolutionary development of the antimicrobial innate immune defense mechanisms, especially ETs against pathogens and may give ideas for future studies on species-specific comparison of innate immune functions.

## Materials and methods

### Obtaining of the blood samples

Blood collection from healthy dogs and wild opossums was approved by the Animal Welfare Commission (Comité de Bioética y Bienestar Animal) under no. FCSA-CBAB-EMV-ACUE-003-2017 at the Escuela de Medicina Veterinaria. The blood collection was conducted in line with the Costa Rica Animal Welfare Law (Ley de Bienestar Animal no. 7451).

The dogs used in the research, belonged to owners who were willing to participate in the study. Samples of dogs were taken from the cephalic vein or saphenous vein and collected without anticoagulant (for serum harvest) or with anticoagulant EDTA (sodium salt of ethylenediaminetetraacetic acid) or heparin tubes. The heparin blood was used for the granulocyte isolation and determination of free DNA in plasma. The EDTA blood was used for the PCR analyses. All tubes were transported at 4°C. Serum was separated by centrifugation (10,000 g for 10 min), and frozen at −20°C. EDTA blood samples were frozen at −20°C until further processing and the blood samples in heparin tubes were immediately analyzed (see determination of free DNA in blood below).

Opossums were captured in special Havahart steel traps and blood was taken at the “Hospital de Especies Menores y Silvestres de la Escuela de Medicina Veterinaria” of the Universidad Nacional of Costa Rica (UNA). All processes were under the direction and care of veterinarians. Prior to anesthesia, a rapid observation of the behavior (attitude) of the opossums was made. Each opossum was given intramuscularly Zoletil 50® (Tiletamine with Zolazepam) at a dose of 3 mg/kg; or Ketamine (20 mg/kg) in combination with Xylazine (4 mg/kg). At each opossum, an initial assessment of the ventilatory (respiration), hemodynamic (pulse) and consciousness states was performed; followed by a general objective examination every 5–10 min to monitor vital parameters. As soon as the animal was anesthetized, the blood sample was taken from the femoral, saphenous or cephalic vein. Blood was collected in different tubes as described for the dog blood and followed the same procedure. After sampling was completed, each individual was kept under veterinary observation in a recovery area or cage, where they were allowed to fully recover from anesthesia before being reinstated into their habitat.

### Study design, sample size, and analyzed population

Blood samples of 8 dogs and 5 opossums were collected. The dogs were from Heredia, they were 2 males (1 German Shepherd and 1 without defined breed) and 6 females (1 Afghan, 1 German Shepherd and 4 without defined breed). All 8 dogs were adults.

The opossums were captured in Heredia. They were 3 males and 2 females. All opossums were clinically observed as healthy and adult.

### Polymerase chain reaction (PCR)

DNA from dog and opossum EDTA blood samples was extracted with DNeasy Blood and Tissue Kit (Qiagen, Hilden, Germany). The extractions were performed according to the protocol recommended by the manufacturers.

A nested PCR was used to detect the presence of *T. cruzi* DNA. A segment of approximately 667 bp of the 18S rRNA gene was amplified (Pinto et al., 2015; Aleman et al., 2017). The primers used in the first round were SSU4_F (5′-TGCCAGCACCCGCGGTAAT-3′) and 18Sq1R (5′-CCACCGACCAAAAGCGGCCA-3′) (Pinto et al., 2015). The primers used in the second round were SSU561F (5′-TGGGATAACAAAGGAGCA-3′) and SSU561R (CTGAGACTGTAACCTCAAAGC) (Noyes et al., 1999).

The PCR reaction was performed in a final volume of 25 μl, adding 1 μl of DNA from the sample, 12.5 μl DreamTaq^TM^ PCR Master Mix 2X (ThermoScientific, USA), 0.5 μl of each of the pair of initiators in each round (16 μM) and 11 μl of nuclease-free water (ThermoScientific, USA) (Noyes et al., 1999). As positive control was used a sample from canine blood previously confirmed by sequencing as positive for *T. cruzi* (Genbank accession number MH045194), Nuclease-free water was used as a negative control (ThermoScientific, USA).

The protocol for nested PCR of the 18S rRNA gene for the detection of *T. cruzi* DNA consists of a touch down gradient protocol, which was used in both rounds and described in Supplemental Table 3 (Murphy and O'Brien, 2007; Aleman et al., 2017).

Electrophoresis of all second PCR products were performed on 1% agarose gels (Noyes et al., 1999), using GelRed to stain the DNA, and run in an electrophoresis chamber at 100 V of 30–40 min. The amplicons that showed a molecular weight of approximately 667 bp were considered positive for *T. cruzi* and sent for sequencing to Macrogen Inc (Seoul, South Korea). The partial sequences obtained were aligned with the BioEdit Sequence Alignment Editor® program (Hall, 1999) and compared using the BLASTn algorithm with the database of the National Center for Biotechnology Information (NCBI).

### Determination of free DNA in blood

Two hundred micro liter fresh taking heparinized blood was diluted 1:2 with PBS and incubated for 2 h at 37°C 5% CO_2_ and every 30 min mixed by hand. Afterwards samples were centrifuged 5 min at 3,500 g and the plasma was collected and stored at −20°C for later analysis with Pico Green assay (See below).

### Pico green assay

For quantification of free DNA, a spectrofluorometric method was conducted using the Quant-iT™ PicoGreen®dsDNA kit (Invitrogen) as previously described (von Köckritz-Blickwede et al., 2010).

### Growth of *T. cruzi*

The local *T. cruzi* ALF strain (GenBank accession number MH020170) isolated from a bug (*Triatoma dimidiata*) was used for these experiments. *T. cruzi* trypomastigotes were grown on monolayers of mouse 3T3 fibroblasts as previously described (Van Voorhis and Eisen, 1989). Briefly, the 3T3 fibroblasts were seeded in a T25 flask and infected with suspension of trypomastigotes adjusted to 1.2 × 10^6^ trypomastigotes / mL (3:1, three trypomastigotes per cell). The cells were incubated at 37°C and 5% CO_2_ in DMEM (Dulbecco's Modified Eagle's Medium) supplemented with 2% fetal bovine serum (FBS). After 24 h incubation non-infective extracellular parasites were washed away with PBS and fresh medium was added. Between 6 and 8 days after infection, the bottles were examined in order to determine amastigotes in the cells, noting the presence or absence of extracellular trypomastigotes. From the culture medium collected from the bottles with infected cells, the parasites were counted in the Neubauer chamber and used to make infections.

### Isolation of granulocytes from didelphine and canine blood

Granulocytes were purified from heparin blood using Biocoll 1077 (Millipore) and hypotonic lysis of erythrocytes. In detail 15 ml fresh blood was diluted with 10 ml PBS. 12.5 ml blood-PBS-mixture was put on the top of 15 ml Biocoll. This gradient was centrifuged 20 min, 400 g with no brake at 20°C. The hypotonic erythrocyte lysis was conducted for 30 s and afterwards stopped. A centrifugation for 7 min with no brake at 280 g and 4°C pelleted the granulocytes. The lysis step was repeated up to 3 times until the pellet was white. Afterwards the granulocytes were counted in a Neubauer chamber and adjusted in RPMI to the cell number as described in the experiments.

### ET induction

Canine and didelphine blood-derived granulocytes were seeded in 48 well plates in a concentration of 2 × 10^5^ cells per well. The final volume per well was 200 μl. The cells were stimulated with *T. cruzi* MOI 2 or 5 and centrifuged for 5 min at 370 g. The cells were incubated for 60 or 180 min at 37°C and 5% CO_2_. Methyl-β-cyclodextrine (CD, final concentration10 mM) and PBS medium alone served as positive and negative controls, respectively.

For the quantification of free DNA cells and stimuli were co-incubated. To determine free DNA at the end of incubation a centrifugation for 5 min at 370 g was conducted and the supernatants collected and stored at −20°C for later analysis with Pico Green assay (see before).

For visualization of ETs and ET quantification by immunofluorescence microscopy in each well a poly-l-lysine coated cover slid was added as described previously (de Buhr et al., 2014). Finally, cells were fixed with 4% paraformaldehyde.

### Immunofluorescence staining

For the staining of ETs samples were permeabilized for 5 min (0.5% Triton X-100; Invitrogen) and blocked for 20 min (blocking buffer with 3% normal donkey serum, 3% cold water fish gelatin, 1% BSA, and 0.05% Tween20 in PBS). Then, the samples were incubated with a mouse monoclonal-antibody against DNA/histone 1 (4.4 μg/ml in blocking buffer, MAB3864; Millipore) for 1 h to visualize the ETs. The secondary staining was performed using a goat anti-mouse DyLight 488-conjugated antibody (1:500; Invitrogen). Staining with aqueous Hoechst 33342 (0.5 mg/ml) was performed for 10 min. After washing, all coverslips were embedded in ProLong® Gold antifade reagent (Invitrogen).

For the co-staining of ETs and neutrophil elastase within ETs, samples were prepared as described above. Then, the samples were incubated with a mouse monoclonal-antibody against DNA/histone 1 (4.4 μg/ml in blocking buffer, MAB3864; Millipore) for 1 h to visualize the ETs and a rabbit anti-neutrophil elastase antibody (1:25 in blocking buffer, Abcam #AB1876 [Cambridge, UK]) in blocking buffer. The secondary staining was performed using a goat anti-mouse Dylight 488-conjugated antibody (1: 500; Invitrogen) or a goat anti-rabbit Alexa 568-conjugated antibody (1: 500; Invitrogen). And stained aqueous Hoechst 33342 and embedded in ProLong® Gold antifade reagent as described above.

### Immunofluorescence microscopy and 3D imaging

Samples were recorded using a Leica TCS SP5 confocal inverted-base fluorescence microscope with a HCX PL APO 40 × 0.75–1.25 oil immersion objective. Settings were adjusted with control preparations using an isotype control antibody. For each sample 6–7 randomly selected images per independent experiment were acquired and used for quantification of the granulocyte number and the induced ET forming cells with an occurrence of a distinct extracellular off-shoot of the cells which is positive for histone-DNA complexes. For the 3D picture and movie, z-stacks were collected and analyzed with LAS X 3D Version 3.1.0 software from Leica. Fifty-six z-stack pictures were used and the background set to gray by standard software settings. The movie was made with the movie maker in the software.

### Statistical analysis

Data were analyzed using Excel 2016 (Microsoft) and GraphPad Prism 7.02 (GraphPad Software). Normal distribution of data was verified by Kolmogorov Smirnov normality test (GraphPad software) prior to statistical analysis. Differences between two groups were determined using an one-tailed paired Student's *t*-test in case of normal distributed data or a one-tailed unpaired Student's *t*-test in case of the NET induction experiment. Outliers were identified with Grubbs test and excluded. Probabilities lower than 0.05 were considered significant (^*^p < 0.05, ^**^p < 0.005, ^***^p < 0.0005, ^****^p < 0.0001), lower or equal than 0.1 as statistically noticeable.

## Author contributions

NdB, GD, and MvK-B: conceived and designed the experiments; NdB, MB, GD, and MJ-S: performed the experiments; NdB, and MB: analyzed the data; NdB, MvK-B, GD, and MB: wrote the manuscript. All authors contributed to manuscript revision, read and approved the submitted version.

### Conflict of interest statement

The authors declare that the research was conducted in the absence of any commercial or financial relationships that could be construed as a potential conflict of interest.
