# Supplementary material for: Extracellular Trap Formation in Response to Trypanosoma cruzi Infection in Granulocytes Isolated From Dogs and Common Opossums, Natural Reservoir Hosts
Source: Front Microbiol. 2018 May 15;9:966. doi: 10.3389/fmicb.2018.00966 (PMC5962733; doi:10.3389/fmicb.2018.00966)
Supplement: Supplementary file 1 [file Data_Sheet_1.pdf]

Supplementary Material

Extracellular trap formation in response to *Trypanosoma cruzi* infection in granulocytes isolated from dogs and common opossums, natural reservoir hosts

Nicole de Buhr[1,2*], Marta C. Bonilla[3], Mauricio Jimenez-Soto[3], Maren von Köckritz-Blickwede[1,2†] Gaby Dolz[3†],

[1]Department of Physiological Chemistry, Department of Infectious Diseases, University of Veterinary Medicine Hannover, Hannover, Germany, [2]Research Center for Emerging Infections and Zoonoses (RIZ), University of Veterinary Medicine Hannover, Hannover, Germany, [3]Escuela de Medicina Veterinaria, Universidad Nacional, Heredia, Costa Rica

†These senior authors contributed equally to this paper.

* CORRESPONDENCE:

Nicole de Buhr

Nicole.de.buhr@tiho-hannover.de

1 Supplementary Methods

DIFF-Quick staining and analysis

Blood smears or smears of isolated granulocytes (20µl) were air dried and stained according to the protocol recommended by the manufacturers with a HAEMA fast stain (DIFF Quick, Labor und Technik Eberhard Lehmann GmbH, Germany). The analysis was conducted with 40-fold magnification and a LEICA light microscope. The purity of the isolated cells was analysed: 100 cells per animal were counted and classified in cell types.

2 Supplementary Figures and Tables

2.1 Supplementary Figures

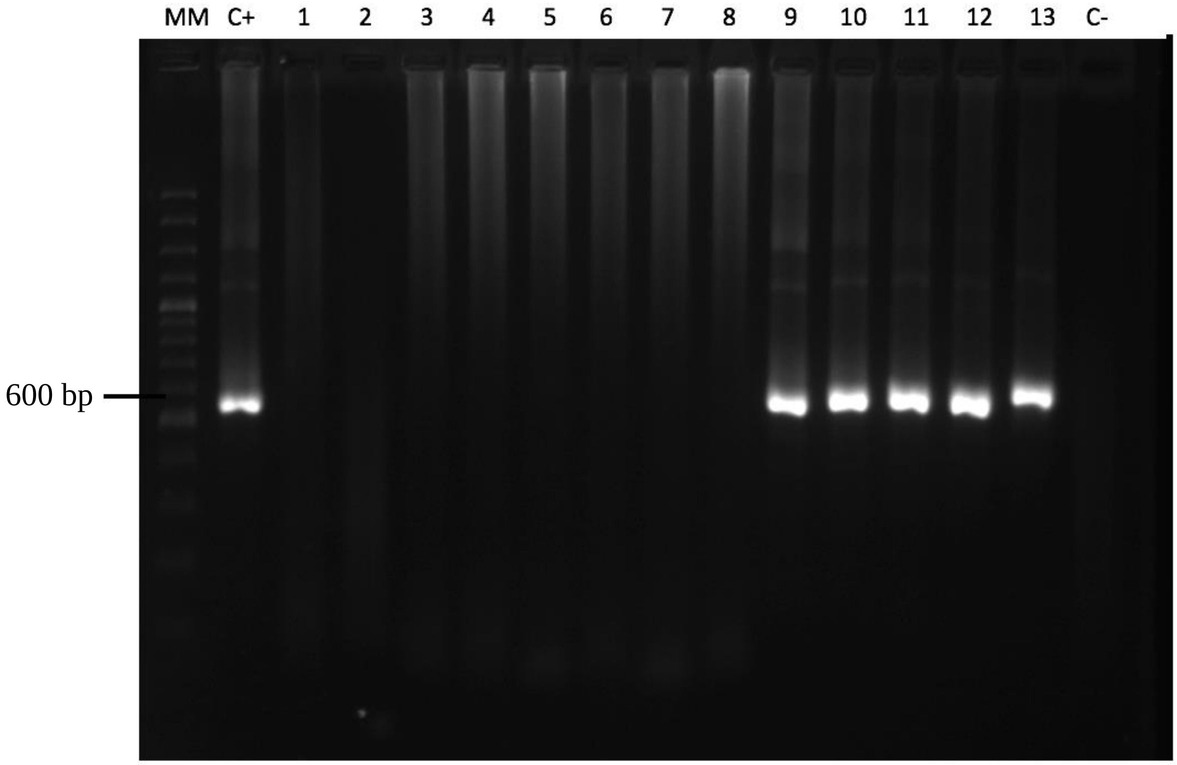

Supplementary Figure 1. Representative 1% agarose gel of the PCR products for *T. cruzi*.
MM: Molecular weight marker, C+: Positive control of *T. cruzi*, 1-8: Canine blood samples, 9-13 Opossum blood samples, C-: Negative control.

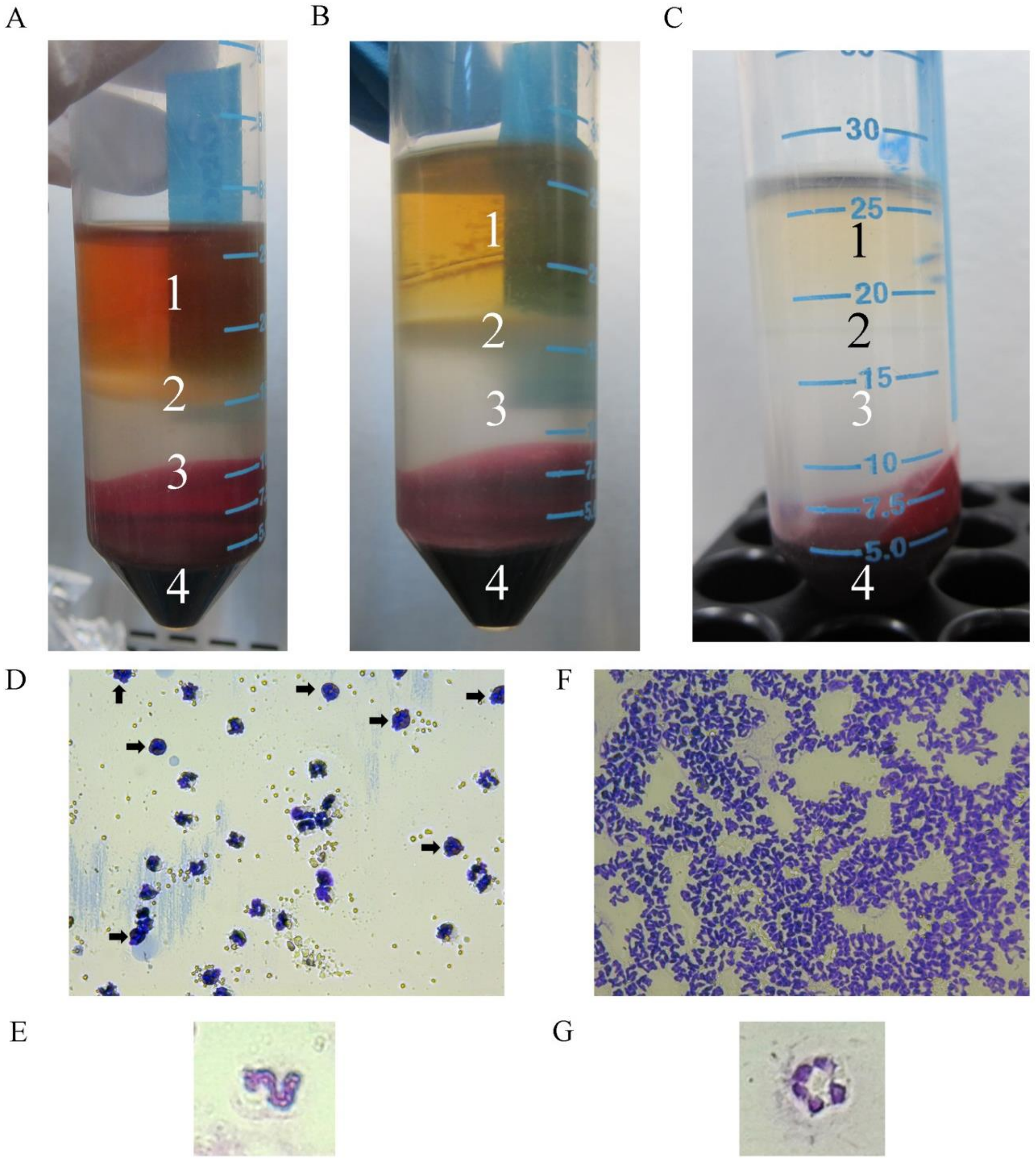

Supplementary Figure 2. Comparison of granulocyte isolation from canine and didelphine blood

The isolation of granulocytes was conducted from 15ml blood using Biocoll density gradient. The result after the first centrifugation step with typical layers is shown: 1 plasma, 2 peripheral blood mononuclear cell (PBMC), 3 Biocoll, 4 erythrocytes and granulocytes. Gradients are representative for A) opossum male, B) opossum female, C) dog female. Granulocytes were stained with DIFF-Quick and analyzed by light microscopy with 40-fold magnification. Images shown are representative for both species, D-E) opossum and F-G) dog. Eosinophil granulocytes are indicated by a black arrow in D.

2.2 Supplementary Tables

number of granulocytes in 15 ml	**dog** (***Canis lupus familaris***)	**common opossum** (***Didelphis marsupialis***)
theoretical number (mean)	1×10^8	7.68×10^7
isolated number (male)	3.48×10^7 (n= 2)	4.8×10^7 (n= 2)
isolated number (female)	1.60×10^7 (n= 6)	5.9×10^7 (n= 2)
isolated number (all)	2.07×10^7 (n= 8)	5.36×10^7 (n= 4)

Supplementary Table 1. Based on the mean of granulocytes / µl [Table 1] the theoretical number of granulocytes in 15 ml blood was calculated.

% in 100 cells after isolation	**dog** (***Canis lupus familaris***)	**common opossum** (***Didelphis marsupialis***)
Neutrophil granulocyte	99	66.5
Eosinophil granulocyte	0	33.25
Basophil granulocyte	0	0
Lymphocyte	0.66	0.25
Monocyte	0.33	0

Supplementary Table 2. To determine the purity of cell population after density gradient, 100 cells were counted after DIFF Quick staining. The mean of 3 dogs and 4 opossums are presented.

**Cycles**	**Denaturation**	**Annealing**	**Elongation**
Initial denaturation	2min/95ºC	-	-
2 cycles	30s/95ºC	30s/60ºC	1min/72ºC
2 cycles	30s/95ºC	30s/58ºC	1min/72ºC
2 cycles	30s/95ºC	30s/56ºC	1min/72ºC
2 cycles	30s/95ºC	30s/54ºC	1min/72ºC
2 cycles	30s/95ºC	30s/52ºC	1min/72ºC
30 cycles	30s/95ºC	30s/50ºC	1min/72ºC
Final elongation	-	-	5min/72ºC

Supplementary Table 3. Touch-down gradient protocol for the nested PCR of the *T. cruzi* 18S rRNA gene.
